# Diversity and Distribution of *nirK*-Harboring Denitrifying Bacteria in the Water Column in the Yellow River Estuary

**DOI:** 10.1264/jsme2.ME13111

**Published:** 2014-03-13

**Authors:** Jing Li, Guangshan Wei, Ningxin Wang, Zheng Gao

**Affiliations:** 1 State Key Laboratory of Crop Biology, College of Life Sciences, Shandong Agricultural University, Taian, Shandong 271018, People’s Republic of China; 2 College of Plant Protection, Shandong Agricultural University, Taian, Shandong 271018, People’s Republic of China

**Keywords:** denitrification, *nirK* gene, Yellow River estuary, water column

## Abstract

We investigated the diversity and community composition of denitrifying bacteria in surface water from the Yellow River estuary. Our results indicated that the diversity of the denitrifying community in freshwater based on the *nirK* gene was higher than that in seawater. Furthermore, phylogenetic analysis suggested that the bacteria community could be distributed into eight clusters (Clusters I to VIII). Redundancy analysis (RDA) revealed that community compositions were related to multiple environment factors, such as salinity and nitrate concentration. The results of the present study have provided a novel insight into the denitrifying community in water columns in estuaries.

Nutrient removal has played an important role in preventing the eutrophication of receiving waters (22). Denitrification, as an effective way to remove nitrogen, is an alternative anaerobic respiration process that removes nitrogen via the microbial stepwise reduction of NO_3_^−^ to gaseous products (NO, N_2_O, N_2_) (5). Nitrite reductase is the rate-limiting enzyme in denitrification, and catalyzes the step from nitrite reduction to nitric oxide. Thus, it has commonly been used as a molecular marker of denitrifying bacteria. Two main classes of functionally-equivalent nitrite reductase have been identified in denitrifying bacteria: a copper-containing NirK enzyme and cytochrome *cd_1_* NirS nitrite reductase (2, 16, 25). The *nirK* gene, which encodes nitrite reductase, has extensively been used in recent years to clarify the composition of the denitrifier community and diversity in the water columns of freshwater and seawater (10, 14, 17, 21).

An estuary is a complex ecosystem that receives extensive river discharges of various terrestrial and anthropogenic materials, such as nutrients and pollutions (9). Terrestrial inputs and physiochemical parameters have been shown to have a marked impact on the diversity and community composition of denitrifying bacteria in the water column of an estuary. The Yellow River estuary is located in the eastern coastal area of China, and high concentrations of nitrogen are a feature in this water column. The importation of nitrogen has recently been shown to be increasing in the Yellow River estuary (13). However, the diversity and community composition of denitrifying bacteria in surface water in the Yellow River estuary remain unknown. In this study, we investigated the diversity and distribution of denitrifying bacteria in the water column in the Yellow River estuary and revealed relationships between the denitrifying community and environmental parameters.

Four samples were collected from the Yellow River estuary on October 21, 2010 (Fig. S1). Samples from sites A and B were taken from freshwater sources, whereas samples from sites C and D were from seawater sources. Water samples at each site were taken in triplicate at a depth of 0.5 m by a water sampler (Wildlife Supply Company, USA). A l L water sample from each site was filtered through 0.22 μm millipore filters. These filters were stored at −80°C until DNA was extracted. The physicochemical variables at each sampling site were measured three times and the average values are shown in Table 1. Significant differences were observed in the salinity in all sampling sites, ranging from 2.3 to 26.7 g L^−1^. The concentrations of total nitrogen, dissolved oxygen, and nitrate were markedly higher in freshwater than in seawater samples. In contrast, the concentrations of chemical oxygen demand were lower in freshwater than in seawater samples.

The genomic DNA of each sample was extracted using the E.N.Z.A.™ Water DNA Kit (Omega, USA) according to the manufacturer’s instructions. Fragments of the *nirK* and *nirS* genes were amplified using the primer pairs F1aCu-R3Cu for *nirK* (6) and nirS1F-nirS6R for *nirS* (3). The PCR amplification conditions are shown in Table S1. No *nirS* PCR products were obtained in any of the four samples by repeated PCR. The purified PCR products were ligated into the pMD18-T simple vector (TaKaRa, Japan), and then transformed into *Escherichia coli* DH5α competent cells to construct the gene libraries. Approximately 80 colonies were selected from the clone library of each sample. The clones in each library were screened by restriction fragment length polymorphism (RFLP). The PCR products (8 μL) were added to 20 μL reactions and incubated at 37°C for 2 h, containing 1 U each of the enzymes *Hae*III and *Msp*I (TaKaRa, Japan) and 2 μL buffer (24). The representative clones were then selected for sequencing in Majorbio Biomedical Technology Co. Ltd. (Shanghai, China).

Amino acid sequences sharing 95% similarity were clustered into a single operational taxonomic unit (OTU_0.95_) by MOTHUR software (18). Phylogenetic trees were constructed by the MEGA 5.1 program (19) using the neighbor-joining method and maximum composite likelihood model. The diversity indexes were calculated by Biodap software (20). Redundancy analysis (RDA) was performed in CANOCO 4.5 for Windows (1).

In total, 229 clones of *nirK* genes were analyzed, with 63, 60, 50 and 56 clones being obtained from sites A, B, C, and D, respectively. The numbers of OTUs in each library were 24, 23, 14, and 14, respectively, and the coverage of each library varied from 77.8% to 92.7% (Table 2). The library of site A had the highest richness based on ACE and Chao1, while the Shannon-Weiner index and Simpson’s index indicated that the diversity of site B was higher than that of other samples. We also found that the rarefaction curves for seawater samples were markedly flatter than those for freshwater samples (Fig. S2). These results revealed that diversity and richness were markedly higher in freshwater than in seawater samples.

The NirK compositions and relative ratios differed among different sampling sites (Fig. 1). OTU30 and OTU36 were the dominant OTUs in sites A and B. Nevertheless, most dominant clones in sites C and D were significantly different from those in sites A and B. The most dominant OTU was OTU16 in site C, followed by OTU17, whereas OTU13 and OTU6 were the dominant OTUs in site D. Detailed data are shown in Table S2. No common OTU was shared in the four sampling sites (Fig. 1 and Fig. S3). Community composition analysis and Venn diagrams revealed that OTU5, OTU30, and OTU36 appeared in sites A, B, and C, but were absent in site D. Furthermore, OTU27 existed in all sampling sites, except for site C. Furthermore, twelve OTUs were shared in more than one site, and 47 OTUs were detected in only one site (Table S2 and Table S3), which indicated the representive OTUs of each site.

A neighbor-joining (NJ) phylogenetic tree based on amino acid sequences was generated from the 59 NirK OTUs in all sampling sites (Fig. 2). The NirK sequences from these samples were grouped into eight Clusters (I to VIII). Cluster I contained sequences from four sampling sites, the reference sequences of which came from various environments including lake water, sewage, and sediment (8, 11, 12). Thus, these results indicated that Cluster I may be a ubiquitous denitrifying group. Clusters IV and VI also contained sequences from all sampling sites (mainly from seawater samples). These sequences were similar to those in two lakes (lakes Plußsee and Schöhsee), the Baltic Sea (10), and the San Francisco Bay estuary (12). Cluster III contained sequences from all sampling sites except site D, which was closely related to clones previously described from activated sludge (7, 15). The main sequences of Clusters II and V were from freshwater sources (sites A and B), and were closely related to those from Lake Kinneret (8), activated sludge (7, 15), and the San Francisco Bay estuary (12). The NirK amino acid sequences deduced from *nirK* gene sequences in the Yellow River estuary were closely related to sequences from different habitats, which indicated that these clones were widely distributed denitrifying bacteria and may be able to flexibly adapt to various environments.

RDA analysis was employed to determine the influence of environmental factors on the *nirK*-harboring denitrifier community (Fig. 3). The first and second dimensions explained 82.9% and 13.4% of the total variance, respectively. RDA analysis revealed that the community compositions of denitrifying bacteria were related to multiple environment factors, such as salinity and nitrate concentration. The results obtained also indicated that Clusters I, II, and V positively correlated with total nitrogen, nitrate, dissolved oxygen, total phosphorus, and pH, and negatively correlated with temperature, salinity, and chemical oxygen demand. The opposite results were obtained for Clusters IV and VI (Fig. 3 and Table S4). The relationship among sampling sites, community composition, and environmental factors indicated that samples from sites A and B were more similar than those from sites C and D. Dang *et al.* (4) reported a relationship between denitrifying bacteria and the surrounding environment in the Jiaozhou Bay, and the different environmental adaptation strategies of various denitrifying bacteria was subsequently proposed. We demonstrated in the present study that salinity significantly influenced the diversity and community composition of denitrifying bacteria. On one hand, we found that the diversity of the denitrifying community inversely correlated with salinity (Table 1 and Table 2). Similar results have also been reported in previous studies on different habitats (17, 23). On the other hand, the community compositions of denitrifiers were distinct due to their salinities (Fig. 2 and Fig. 3). This result may have been caused by the selection effect of salinity. In a previous study, denitrifying communities in the freshwater of Lake Kinneret were shown to differ from those of marine habitats, suggesting the differentiation of marine and freshwater denitrifying bacteria (8). These results revealed that environmental factors may play critical roles in controlling the diversity and spatial distribution of the denitrifying community.

In summary, this study showed that denitrifying bacteria existed in surface water and were very diverse in the Yellow River estuary. The community compositions and relative ratios of bacterial communities markedly changed with the different sampling sites. To the best of our knowledge, this is the first study to examine the denitrifying bacteria community in the Yellow River estuary, and the results obtained have provided a novel insight into the denitrifying community in the estuary, especially in surface water.

The representative sequences of *nirK* fragments reported in this study have been deposited in GenBank under the accession numbers KF143898 to KF144045.

## Supplementary material



## Figures and Tables

**Fig. 1:**
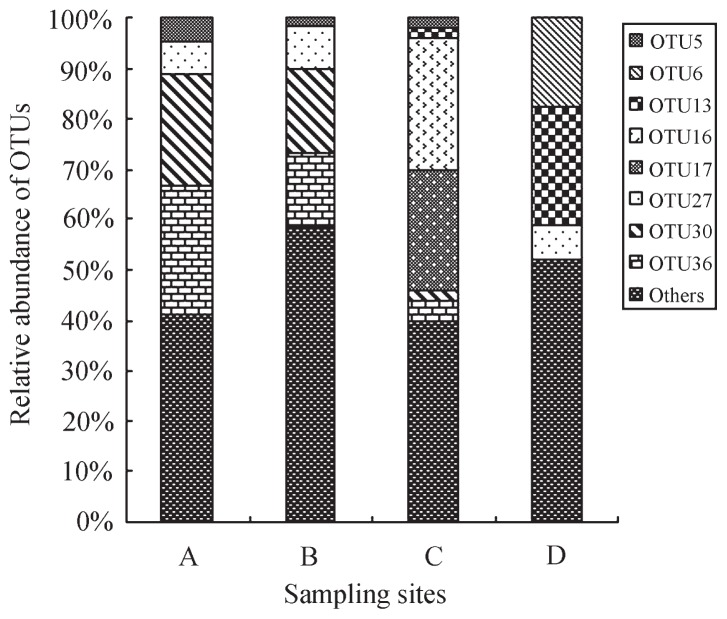
Relative abundance of OTUs in sampling sites A, B, C, and D.

**Fig. 2:**
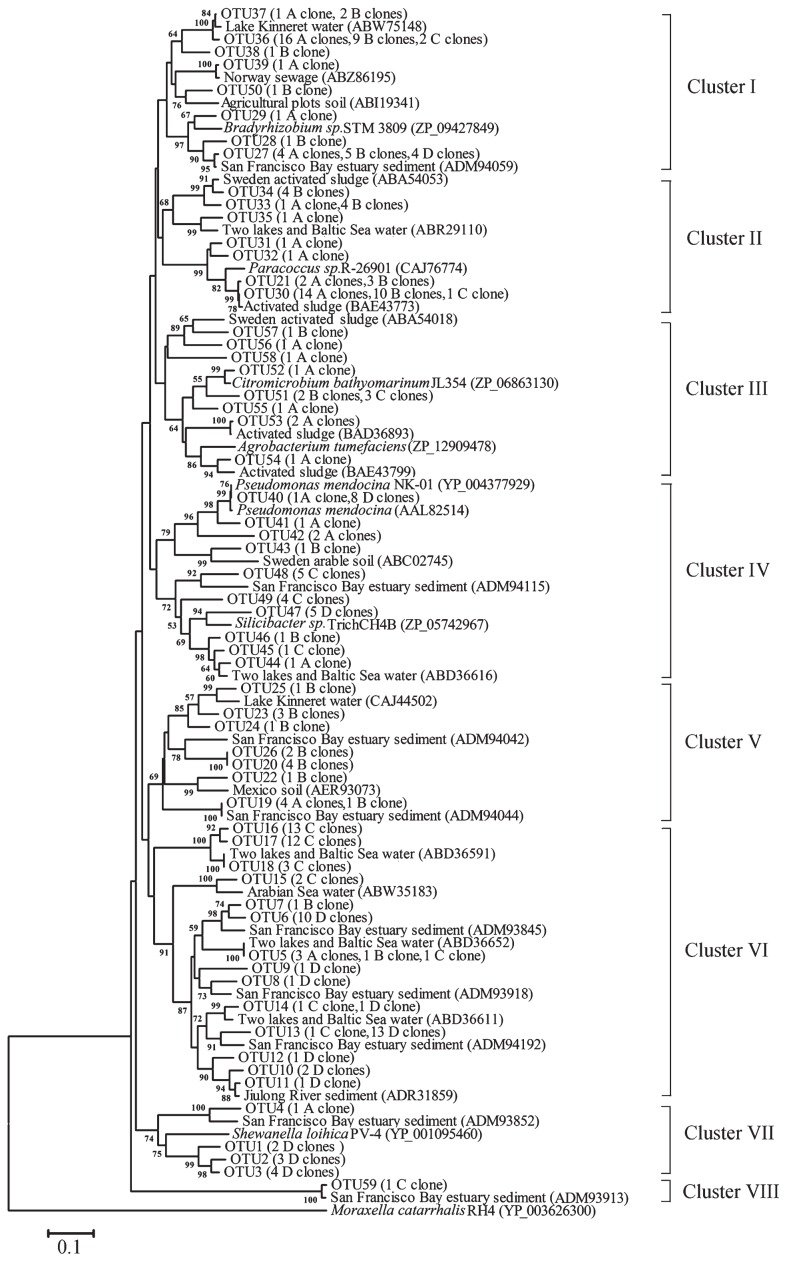
Phylogenetic analysis of denitrifying bacteria based on NirK sequences obtained in the Yellow River estuary. A 5% cut-off in the amino acid sequence was used to define OTUs by MOTHUR. The neighbor-joining method was used and bootstrap analysis was performed with 1,000 replications. Bootstrap values above 50 were indicated at branch points. The *aniA* gene from *Moraxella catarrhalis* (YP_003626300) was used as an outgroup.

**Fig. 3:**
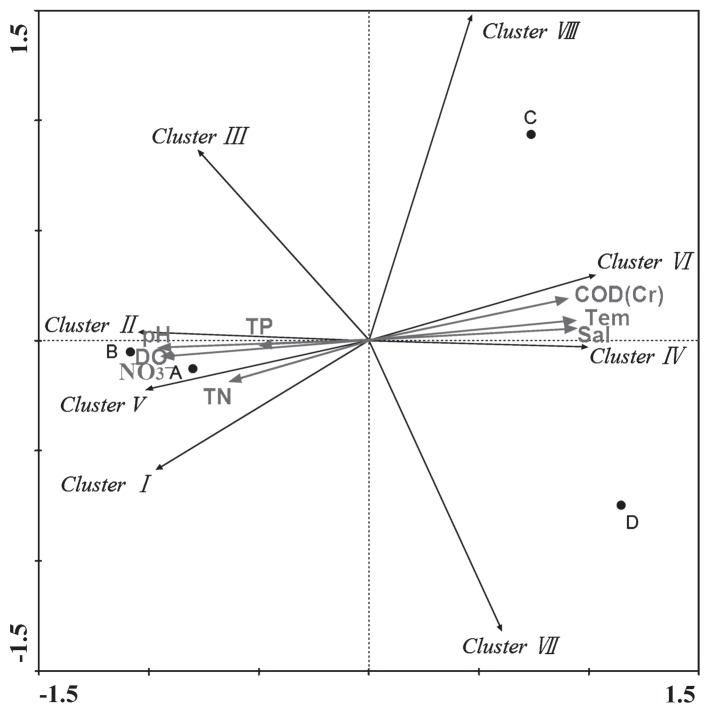
The RDA ordination plot for the relationship between the distribution of Clusters and environmental factors in the Yellow River estuary. Correlations between clusters or environmental factors and RDA axes are represented by the length and angle of arrows. A, B, C, and D represent the four sampling sites.

**Table 1:** Physicochemical properties of water column samples collected from the Yellow River estuary

Sample	Tem^a^ (°C)	pH	Sal^b^ (g L^−1^)	TN^c^ (mg L^−1^)	NO_3_^−^-N^d^ (mg L^−1^)	TP^e^ (mg L^−1^)	DO^f^ (mg L^−1^)	COD^g^ (mg L^−1^)
A	13.9	8.38	2.3	11.0*	2.36*	0.072	9.5	20.2
B	13.9	8.45	3.0	5.7	3.35*	0.115	9.5	18.2
C	14.1	8.14	24.7*	3.1	0.11	0.079	7.6	63.0*
D	14.1	8.10	26.7*	4.5	0.04	0.079	7.5	53.6*

a , temperature;

b , salinity;

c , total nitrogen;

d , nitrate;

e , total phosphorous;

f , dissolved oxygen;

g , chemical oxygen demand;

* , significant differences (P<0.05) according to Duncan’s multiple range test using SAS version 9.1 software.

**Table 2:** Community diversity and predicted richness of NirK sequences from each sampling site

Sampling site	No. of clones	No. of OTUs^a^	*C*(%)^b^	*S*_ACE_^c^	*S*_Chao1_^d^	*D*^e^	*H*^f^	*J*^g^
A	63	24	77.8	59.0	55.1	0.117	2.56	0.80
B	60	23	78.3	46.3	49.0	0.067	2.79	0.89
C	50	14	88.0	20.1	19.8	0.137	2.19	0.83
D	56	14	92.7	19.0	17.4	0.116	2.27	0.86

a , Number of OTUs defined by the furthest neighbor algorithm in MOTHUR at 95% similarity;

b , Coverage=l− (*n_i_*/*N*)×100, where *n_i_* refers to the number of clones appearing only once in each library and *N* refers to the total number of clones in the library;

c , Richness-based coverage estimator;

d , the estimated richness;

e , Simpson’s index;

f , Shannon-Wiener index;

g , Evenness index.

## References

[1] ter Braak CJF, Smilauer P (2002). CANOCO Reference Manual and CanoDraw for Windows User’s Guide: Software for Canonical Community Ordination (version 4.5).

[2] Braker G, Zhou JZ, Wu LY, Devol AH, Tiedje JM (2000). Nitrite reductase genes (*nirK* and *nirS*) as functional markers to investigate diversity of denitrifying bacteria in Pacific northwest marine sediment communities. Appl Environ Microbiol.

[3] Brettar I, Moore ER, Höfle MG (2001). Phylogeny and abundance of novel denitrifying bacteria isolated from the water column of the central Baltic Sea. Microb Ecol.

[4] Dang H, Wang C, Li J, Li T, Tian F, Jin W, Ding Y, Zhang Z (2009). Diversity and distribution of sediment *nirS*-encoding bacterial assemblages in response to environmental gradients in the eutrophied Jiaozhou Bay, China. Microb Ecol.

[5] Dell EA, Bowman D, Rufty T, Shi W (2010). The community composition of soil-denitrifying bacteria from a turfgrass environment. Res Microbiol.

[6] Hallin S, Lindgren PE (1999). PCR detection of genes encoding nitrite reductase in denitrifying bacteria. Appl Environ Microbiol.

[7] Hallin S, Throbäck IN, Dicksved J, Pell M (2006). Metabolic profiles and genetic diversity of denitrifying communities in activated sludge after addition of methanol or ethanol. Appl Environ Microbiol.

[8] Junier P, Kim OS, Witzel KP, Imhoff JF, Hadas O (2008). Habitat partitioning of denitrifying bacterial communities carrying *nirS* or *nirK* genes in the stratified water column of Lake Kinneret, Israel. Aquat Microb Ecol.

[9] Kennish MJ (2002). Environmental threats and environmental future of estuaries. Environ Conserv.

[10] Kim OS, Imhoff JF, Witzel KP, Junier P (2011). Distribution of denitrifying bacterial communities in the stratified water column and sediment-water interface in two freshwater lakes and the Baltic Sea. Aquat Ecol.

[11] Mao Y, Bakken LR, Zhao L, Frostegård Å (2008). Functional robustness and gene pools of a wastewater nitrification reactor: comparison of dispersed and intact biofilms when stressed by low oxygen and low pH. FEMS Microbiol Ecol.

[12] Mosier AC, Francis CA (2010). Denitrifier abundance and activity across the San Francisco Bay estuary. Environ Microbiol Rep.

[13] Mou X, Sun Z, Wang L, Wang C (2011). Nitrogen cycle of a typical Suaeda salsa marsh ecosystem in the Yellow River estuary. J Environ Sci.

[14] Oakley BB, Francis CA, Roberts KJ, Fuchsman CA, Srinivasan S, Staley JT (2007). Analysis of nitrite reductase (*nirK* and *nirS*) genes and cultivation reveal depauperate community of denitrifying bacteria in the Black Sea suboxic zone. Environ Microbiol.

[15] Osaka T, Yoshie S, Tsuneda S, Hirata A, Iwami N, Inamori Y (2006). Identification of acetate-or methanol-assimilating bacteria under nitrate-reducing conditions by stable-isotope probing. Microb Ecol.

[16] Philippot L, Hallin S, Schloter M (2007). Ecology of denitrifying prokaryotes in agricultural soils. Adv Agron.

[17] Santoro AE, Boehm AB, Francis CA (2006). Denitrifier community composition along a nitrate and salinity gradient in a coastal aquifer. Appl Environ Microbiol.

[18] Schloss PD, Westcott SL, Ryabin T (2009). Introducing MOTHUR: open-source, platform-independent, community-supported software for describing and comparing microbial communities. Appl Environ Microbiol.

[19] Tamura K, Peterson D, Peterson N, Stecher G, Nei M, Kumar S (2011). MEGA5: molecular evolutionary genetics analysis using maximum likelihood, evolutionary distance, and maximum parsimony methods. Mol Biol Evol.

[20] Thomas G, Clay D, Magurran A (2000). Biodap-ecological, diversity and its measurement.

[21] Yan T, Fields MW, Wu L, Zu Y, Tiedje JM, Zhou J (2003). Molecular diversity and characterization of nitrite reductase gene fragments (*nirK* and *nirS*) from nitrate- and uranium-contaminated groundwater. Environ Microbiol.

[22] Yao S, Ni J, Chen Q, Borthwick AG (2013). Enrichment and characterization of a bacteria consortium capable of heterotrophic nitrification and aerobic denitrification at low temperature. Bioresour Technol.

[23] Yoshie S, Noda N, Tsuneda S, Hirata A, Inamori Y (2004). Salinity decreases nitrite reductase gene diversity in denitrifying bacteria of wastewater treatment systems. Appl Environ Microbiol.

[24] Zhang R, Thiyagarajan V, Qian PY (2008). Evaluation of terminal-restriction fragment length polymorphism analysis in contrasting marine environments. FEMS Microbiol Ecol.

[25] Zumft WG (1997). Cell biology and molecular basis of denitrification. Microbiol Mol Biol Rev.

