# Supplementary material for: Diversity and Distribution of nirK-Harboring Denitrifying Bacteria in the Water Column in the Yellow River Estuary
Source: Microbes Environ. 2014 Mar 13;29(1):107–10. doi: 10.1264/jsme2.ME13111 (PMC4041238; doi:10.1264/jsme2.ME13111)
Supplement: Supplementary file 1 [file 29_107_s1.pdf]

Fig. S1. A map of sampling sites in the Yellow River estuary.

Fig. S1. ***First author: JING LI***

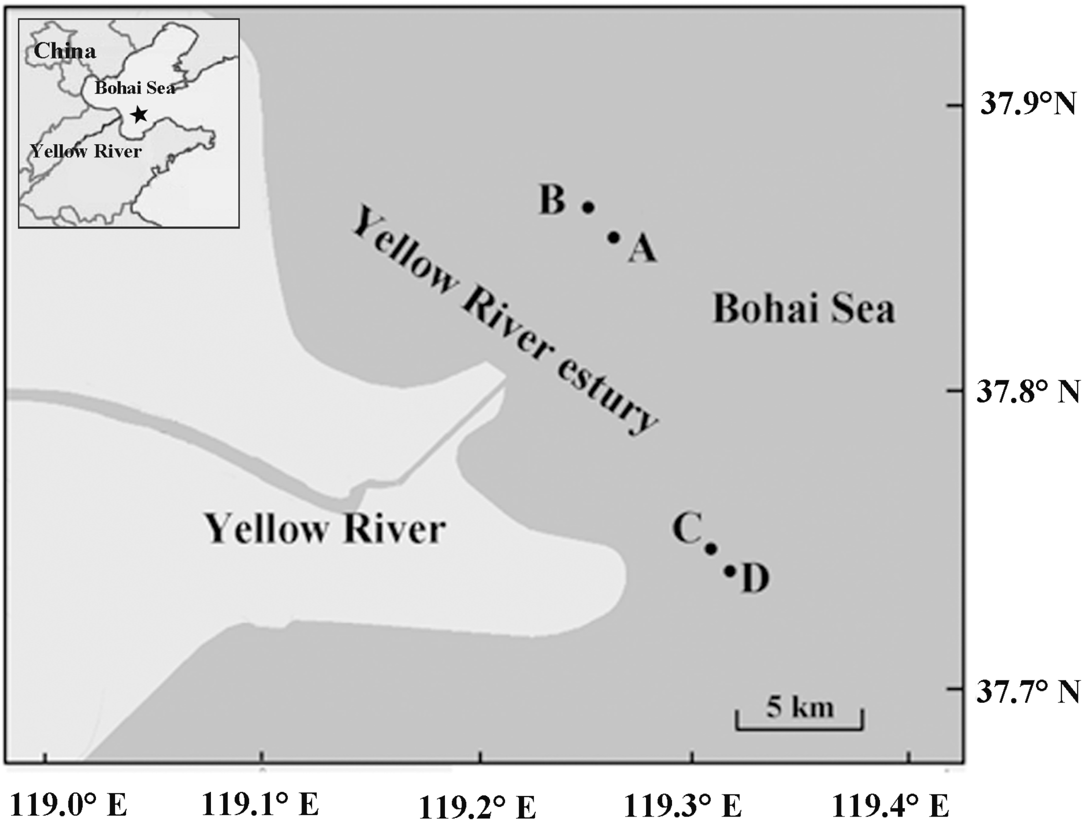

Fig. S2. Rarefaction curves for the *nirK* clone libraries of each sampling site. A 5% cut-off in the amino acid sequence was used to define OTUs.

Fig. S2. ***First author: JING LI***

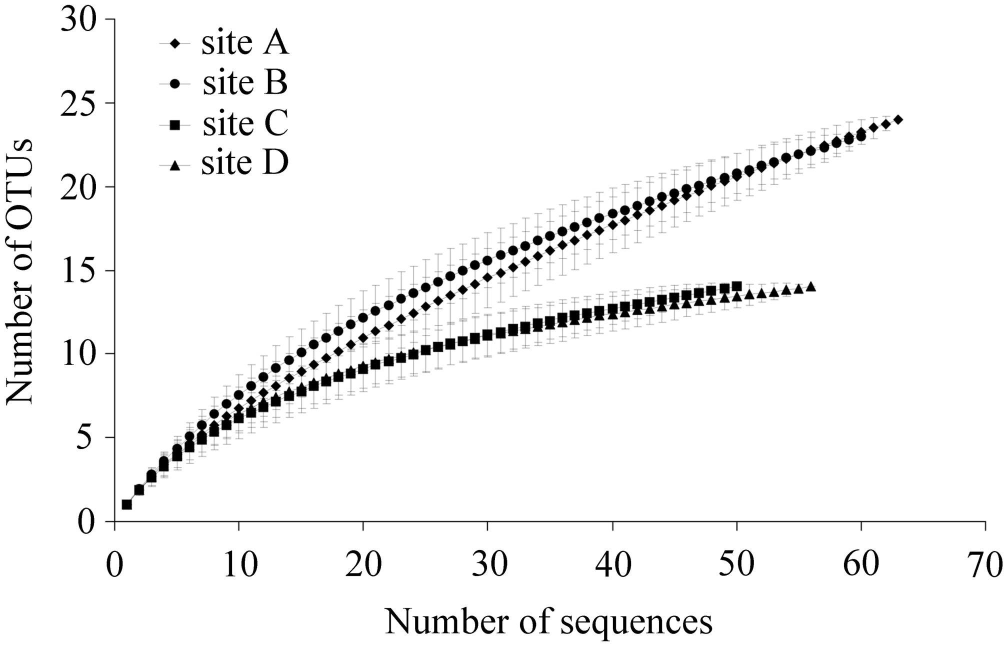

Fig. S3. Venn diagram based on OTUs in sampling sites A, B, C, and D. The numbers represent the number of OTUs that were shared among the different sampling sites.

Fig. S3. ***First author: JING LI***

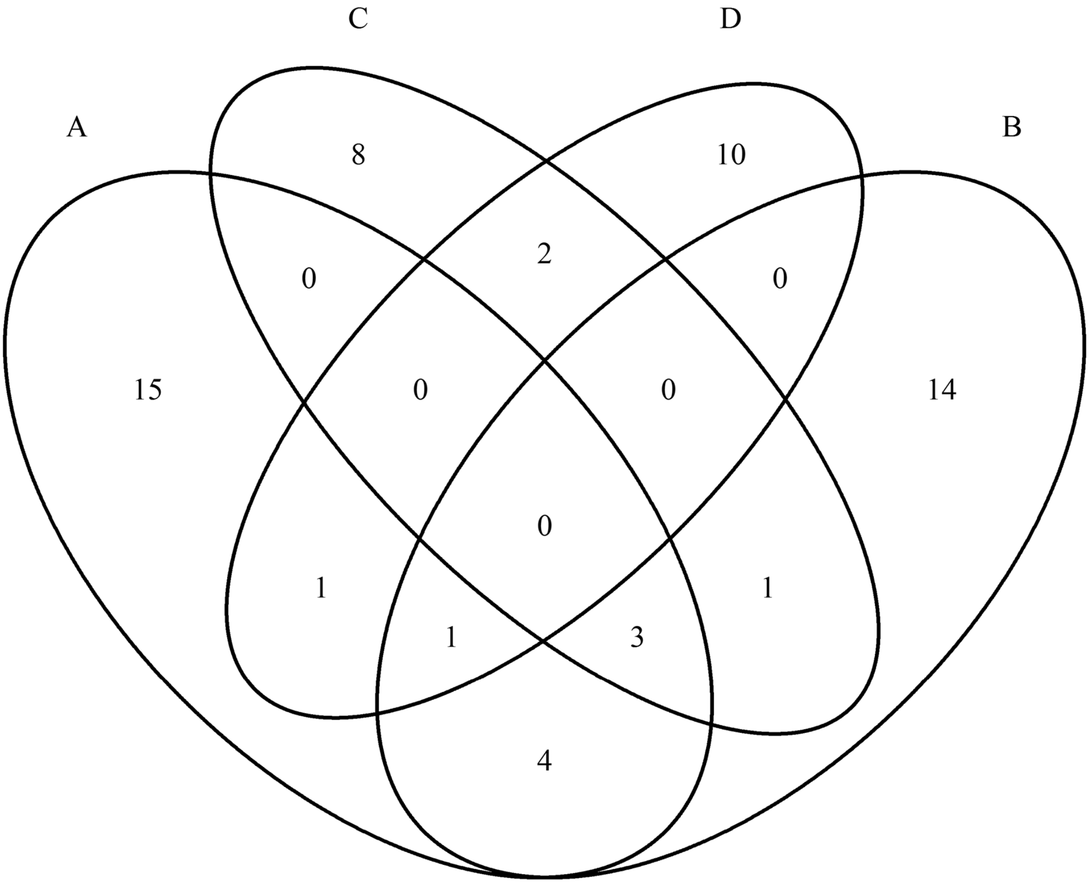

Table S1. PCR amplification conditions

Primers pair	Amplification conditions
F1aCu, R3Cu	8 min at 94°C; 40 s at 94°C, 40 s at 53°C, 50 s at 72°C for 35 cycles; 10 min at 72°C
nirS1F, nirS6R	10 min at 94°C; 40 s at 94°C, 40 s at 52°C, 1 min at 72°C for 35 cycles; 10 min at 72°C

Table S2. Relative abundance of OTUs in sampling site A, B, C and D

OTU	Sampling site			
	A (%)	B (%)	C (%)	D (%)
OTU1	0	0	0	3.57
OTU2	0	0	0	5.36
OTU3	0	0	0	7.14
OTU4	1.59	0	0	0
OTU5	4.76	1.67	2.00	0
OTU6	0	0	0	17.86
OTU7	0	1.67	0	0
OTU8	0	0	0	1.79
OTU9	0	0	0	1.79
OTU10	0	0	0	3.57
OTU11	0	0	0	1.79
OTU12	0	0	0	1.79
OTU13	0	0	2.00	23.21
OTU14	0	0	2.00	1.79
OTU15	0	0	4.00	0
OTU16	0	0	26.00	0
OTU17	0	0	24.00	0
OTU18	0	0	[illegible].00	0
OTU19	6.35	1.67	0	0
OTU20	0	6.67	0	0
OTU21	3.17	5.00	0	0
OTU22	0	1.67	0	0
OTU23	0	5.00	0	0
OTU24	0	1.67	0	0
OTU25	0	1.67	0	0
OTU26	0	3.33	0	0
OTU27	6.35	8.33	0	7.14
OTU28	0	1.67	0	0
OTU29	1.59	0	0	0
OTU30	22.22	16.67	2.00	0
OTU31	1.59	0	0	0
OTU32	1.59	0	0	0
OTU33	1.59	6.67	0	0
OTU34	0	6.67	0	0
OTU35	1.59	0	0	0
OTU36	25.40	15.00	4.00	0
OTU37	1.59	3.33	0	0
OTU38	0	1.67	0	0
OTU39	1.59	0	0	0
OTU40	1.59	0	0	14.29

OTU41	1.59	0	0	0
OTU42	3.17	0	0	0
OTU43	0	1.67	0	0
OTU44	1.59	0	0	0
OTU45	0	0	2	0
OTU46	0	1.67	0	0
OTU47	0	0	0	8.93
OTU48	0	0	10	0
OTU49	0	0	8	0
OTU50	0	1.67	0	0
OTU51	0	3.33	6	0
OTU52	1.59	0	0	0
OTU53	3.17	0	0	0
OTU54	1.59	0	0	0
OTU55	1.59	0	0	0
OTU56	1.59	0	0	0
OTU57	0	1.67	0	0
OTU58	1.59	0	0	0
OTU59	0	0	2	0

Table S3. Distribution of the unique OTUs in each sampling site

Sampling site	Distribution of the unique OTUs	Total
A	4,29,31,32,35,39,41,42,44,52,53,54,55,56,58	15
B	7,20,22,23,24,25,26,28,34,38,43,46,50,57	14
C	15,16,17,18,45,48,49,59	8
D	1,2,3,6,8,9,10,11,12,47	10

Table S4. Correlation coefficients between environmental factors and species axis

Environmental factor	AX1	AX2
pH	-0.997	-0.053
Sal[a]	0.988	0.094
DO[b]	-0.988	-0.119
TN[c]	-0.662	-0.305
NO_3^--N[d]	-0.981	-0.124
TP[e]	-0.517	-0.033
COD[f]	0.948	0.316
Tem[g]	0.983	0.155

a, salinity; b, dissolved oxygen; c, total nitrogen; d, nitrate; e, total phosphorous; f, chemical oxygen demand; g, temperature.
